# Investigating size and surface modification to optimise the delivery of nanodiamonds to brain glial cells

**DOI:** 10.1186/s11671-025-04335-2

**Published:** 2025-08-20

**Authors:** Manami Takahashi, Ayaka Takada, Chihiro Suzuki, Kiichi Kaminaga, Masaki Yoshioka, Mariko Handa, Jeff Kershaw, Hiroshi Abe, Takeshi Ohshima, Ryuji Igarashi, Hiroyuki Takuwa

**Affiliations:** 1 Quantum Neuromapping and Neuromodulation Team, Institute for Quantum Life Science, National Institutes for Quantum Science and Technology, 4-9-1 Anagawa, Inage-ku, Chiba, 263-8555 Japan; 2 Department of Quantum Life Science, Graduate School of Science and Engineering, Chiba University, 1-33 Yayoi-cho, Inage-ku, Chiba, 263-0022 Japan; 3 Future Quantum Sensors Team, Institute for Quantum Life Science, National Institutes for Quantum Science and Technology, 4-9-1 Anagawa, Inage-ku, Chiba, 263-8555 Japan; 4 Department of Neurological Surgery, Graduate School of Medicine, Chiba University, 1-8-1 Inohana, Chuo-ku, Chiba, 260-8670 Japan; 5 Department of Molecular Imaging and Theranostics, Quantum Life and Medical Science Directorate, National Institutes for Quantum Science and Technology, 4-9-1 Anagawa, Inage-ku, Chiba, 263-8555 Japan; 6 Quantum Materials and Applications Research Center, Takasaki Institute for Advanced Quantum Science, National Institutes for Quantum Science and Technology, 1233 Watanuki-machi, Takasaki City, 370-1292 Gunma Japan; 7 Department of Materials Science, Tohoku University, 6-6-02 Aramaki-Aza- Aoba, Aoba-ku, Sendai City, 980-8579 Miyagi Japan; 8 School of Life Science and Technology, Institute of Science Tokyo, 2-12-1 Ookayama, Meguro-ku, Tokyo, 152-8550 Japan

## Abstract

Nanodiamonds (NDs) with nitrogen-vacancy (NV) defects have garnered attention as promising nano-quantum sensors due to their high photostability, low biotoxicity, and ability to measure intracellular parameters such as temperature, magnetic fields, and electric fields. While NDs have been extensively studied in in vitro systems, their application in vivo remains underdeveloped. Efficient delivery of NDs to specific cells within biological tissues remains a critical challenge for advancing their applications in the life sciences. In this study, we investigated the intracellular uptake of NDs by glial cells (microglia and astrocytes) in the brain. Twelve types of NDs, differing in size (50 nm, 150 nm, 250 nm, and 350 nm) and surface modification (COOH, HPG and HPG-COOH), were locally injected into the brain parenchyma of mice. The intracellular uptake of NDs was assessed using immunostaining and confocal microscopy. Microglia preferentially internalized HPG-modified NDs. HPG-modified NDs also exhibited high diffusivity, facilitating interactions with surrounding microglia and enhancing uptake efficiency. In contrast, COOH-modified NDs were more efficiently internalized by astrocytes than HPG-modified NDs. This suggests that COOH-modified NDs tend to remain at the local injection site, where inflammation induced by tissue damage may have enhanced the phagocytic activity of astrocytes. These findings demonstrate that the uptake characteristics of NDs differ by cell type. HPG-modified NDs, are optimal for microglia, while COOH-modified NDs, are more suitable for astrocytes. It is anticipated that the results of this study will act as an important guide for the use of NDs as nano-quantum sensors in living brain tissues.

**Supplementary Information:**

The online version contains supplementary material available at 10.1186/s11671-025-04335-2.

## Introduction

Carbon nanoparticles known as nanodiamonds (NDs) have recently gained attention for possible applications in the life sciences. The use of NDs in biological applications has been advancing in in vitro systems. In particular, NDs with nitrogen-vacancy (NV) defect centres can be used in vitro to measure intracellular temperature, magnetic fields, and electric fields through optically detected magnetic resonance (ODMR) [1–3]. NDs with specific surface modifications can also be used to measure parameters such as pH and reactive oxygen species [4, 5]. Due to their ability to function as sensors even at the nanoscale, NDs with NV centres are referred to as nano-quantum sensors [6]. NDs have low biotoxicity and exhibit high photostability, so they are resistant to photobleaching [2, 7]. The biocompatible properties of NDs make them highly promising for intracellular applications in vivo, allowing multi-parameter measurement of the microenvironment within organelles. Some previous applications of quantum sensors to living organisms has been reported. For example, ODMR has been performed on NDs introduced into live *C*. elegans after irradiating them with lasers and microwaves, enabling the detection of physiological temperature changes within tissues [8]. NDs have also been successfully introduced into the bodies of mice post-mortem to perform subcutaneous temperature measurement [9]. It is anticipated that NDs will contribute to the elucidation of various biological mechanisms.

Real-time measurements of neural activity in the brain and other physiological processes are often essential, so there is a high demand for in vivo measurement techniques. The brain is composed of neurons, glial cells and blood vessels, and understanding the various roles of these cells is not only important for neuroscience, but also for elucidating the mechanisms of brain diseases such as neurodegenerative disorders, stroke, and depression. The ability to quantitatively measure multiple physicochemical parameters in brain cells in vivo using nano-quantum sensors would be highly beneficial for both neuroscience and brain disease research. However, a prerequisite for measurement is the development of reliable nanodiamond intracellular delivery methods.

In this study, we investigated methods to improve the introduction of NDs into glial cells in vivo. Glial cells such as microglia and astrocytes are known to play central roles in brain disease pathology [10, 11], making the optimization of ND delivery to those cells highly relevant for a wide range of applications. It is known that foreign materials stimulate phagocytic activity in glial cells [12–14], so it can be expected that NDs will be spontaneously taken up in quantities sufficient for measurement [15]. However, microglia and astrocytes exhibit different phagocytic capacities, with astrocytes generally displaying lower phagocytic activity unless under inflammatory conditions [16]. It has also been reported that microglia engulf neuronal cell bodies containing abnormal tau aggregates, whereas astrocytes preferentially engulf axons, indicating differences in their phagocytic targets [12]. Additionally, highly phagocytic cells such as macrophages and microglia increase their intracellular uptake of nanoparticles depending on the duration of contact [17].

Taking into consideration the differences in glial cell phagocytic characteristics, we formulated the following hypotheses regarding the optimal intracellular uptake of NDs introduced into the brain:

1. Microglia, which exhibit high phagocytic activity, are more likely to engulf NDs when they have a greater chance of encountering them. Therefore, high dispersibility of NDs within the brain parenchyma after local injection may enhance their uptake.
2. In contrast, astrocytes have lower phagocytic activity, so they are more likely to internalize NDs via endocytosis when exposed to a high local concentration. Thus, NDs that remain concentrated at the injection site rather than dispersing may be more effectively taken up by astrocytes.

To test these hypotheses, we prepared 12 types of NDs by combining four different sizes with three different surface modifications. The NDs were locally injected into mouse brain, and after three days the brains were extracted, sectioned, and immunostained to label glial cells. The intracellular uptake rate of NDs by each cell type was then analysed.

## Materials and methods

### Animal preparation

This study used adult male C57BL/6 J mice (7 months old, Japan SLC, N = 12). The mice were housed under a 12-h light/dark cycle with ad libitum access to standard chow and water. All animal care and handling procedures followed the Japan National Research Council’s Guide for the Care and Use of Laboratory Animals. The experimental protocol was approved by the Animal Ethics Committee of the National Institutes for Quantum Science and Technology.

### Production of fluorescent NDs

Type Ib nanodiamond powders (50 nm: PUREON, 150, 250, 350 nm: Element-Six) synthesized by the HPHT method, were irradiated with a 2-MeV proton beam at a dose of 4 X 10^18^ cm^2^, followed by thermal annealing in vacuum at 800 °C for 2 h and air oxidation at 550 °C for 2 h.

- **ND-COOH**: Air Oxidated Fluorescent NDs were treated with an H2SO4:HNO3 (3:1 v/v) mixture at 330 °C for 1 h, a 0.1 M NaOH solution for 4 h at 90 °C, and a 0.1 M HCl solution for 2 h at 90 °C.
- **ND-HPG**: ND-COOH was dispersed in glycidol, sonicated, and stirred at 140 °C for 1 h under atmospheric conditions. The resulting gel was diluted with Milli-Q water and methanol (1:1, v/v), then centrifuged for 30 min to collect ND-HPG. Subsequently, the nanodiamond particles were washed three times by centrifugation to remove free polyglycerol.
- **ND-HPG**-COOH: ND-HPG particles were mixed with succinic anhydride in pyridine at 70 °C for 1.5 h under an argon atmosphere. The reactant gel was diluted with milli-Q: methanol (1:1, v/v) and centrifuged for 30 min to collect ND-HPG-COOH. The particles were then washed three times using centrifugation.

The size of the nanodiamond particles with each surface chemical modification was evaluated using dynamic light scattering (DLS, Zeta-sizer Ultra (Spectris)) (Figure S1).

### Stereotaxic injection

Mice were anesthetized using a nose cone with 1.5% isoflurane and secured in a stereotaxic frame. Rectal temperature was maintained at 36 °C using a warming pad (ATC-210, Unique Medical, Tokyo, Japan). A midline incision was made to expose the skull, and craniotomies (~ 0.5 mm in diameter) were performed at the following bregma-referenced sites:

- Anteroposterior (AP): –1.25 mm, –2.0 mm, –2.75 mm.
- Mediolateral (ML): ± 2.5 mm from the midline.
- Dorsoventral (DV): –0.4 to –0.5 mm from the dura.

The cerebral cortex was chosen as the injection site because it is anatomically superficial and allows for stable and reproducible stereotaxic injections.

The NDs solutions (ND-COOH, ND-HPG and ND-COOH-HPG; 50 mg/mL) were sonicated before injection. A glass micropipette (tip diameter of a few micrometers) was used to inject 0.5 µL of solution into each site at a rate of 0.25 µL/min. To minimize reflux along the injection path, the pipette was left in place for 3 min before being slowly withdrawn. The incision site was sutured and sealed with cyanoacrylate adhesive. Following recovery, mice were returned to their home cages. The brains were harvested for analysis three days post-injection.

### Fluorescent immunohistochemistry

Three days after ND injection, mice were deeply anesthetized and perfused transcardially with 4% paraformaldehyde (PFA) in phosphate-buffered saline (PBS). The extracted brains were post-fixed in 4% PFA/PBS at 4 °C overnight. After washing with PBS, 100-µm-thick coronal brain sections were prepared using a vibratome (VT1200S, Leica). Sections were blocked at room temperature for 30 min in PBS containing 4% bovine serum albumin (BSA), 2% horse serum, and 0.25% Triton X-100. The sections were then incubated overnight at 4 °C with the following primary antibodies: Iba1 polyclonal antibody (1:2000, Wako, cat# 019–19741), GFAP monoclonal antibody (2.2B10) (1:2500, Thermo Fisher, cat# 130,300). After washing with PBS, the sections were incubated for 2 h at room temperature with Alexa Fluor 488-conjugated secondary antibodies (1:500, Invitrogen). The sections were mounted using Aqua-Poly/Mount (Polysciences) to prevent fluorescence fading.

The specificity of the Iba1 antibody for microglia has been well established in previous studies [18, 19], showing selective labeling of microglial cells without cross-reactivity to other cell types. Similarly, GFAP is an intermediate filament protein predominantly expressed in astrocytes, and the monoclonal antibody used specifically recognizes astrocytes and is widely validated for immunohistochemical applications [20, 21]. These antibodies were chosen based on their proven reliability and specificity to accurately identify microglia and astrocytes in brain tissue. In addition to using validated commercial antibodies, we confirmed the specificity of Iba1 and GFAP labeling based on the characteristic morphology and spatial localization of the labeled cells in the brain parenchyma.

### Confocal microscopy imaging

Immunofluorescence images were acquired with a confocal laser scanning microscope (LSM 980, Carl Zeiss). Excitation was performed using 488 nm and 543 nm solid-state lasers, and fluorescence emission signals were detected using the following bandpass filters: 490–543 nm (for 488 nm excitation), 562–696 nm (for 543 nm excitation). To accurately separate overlapping fluorescence signals, spectral imaging and linear unmixing were performed using the ZEISS image processing platform (Figure S2). During image acquisition, the Lambda mode was employed to capture a spectral dataset covering the entire emission spectrum of the fluorophores. The spectral information was computationally separated using a linear unmixing algorithm with reference spectra for each fluorophore. Images were acquired at a resolution of 1024 × 1024 pixels, with a pixel size of 103.6 nm in the x/y plane. Optical z-stack images were obtained at a step size of 250 nm, and collected in order from the bottom to the top of the sample. Image analysis was performed using Fiji (NIH) [22], and maximum intensity projections were generated from the acquired z-stack images. For each animal and brain region, 4–6 sections were analysed. Cell quantification was also conducted with Fiji, employing manual cell counting and a randomized stereological estimation method, and performed by researchers blinded to the experimental conditions.

## Results

### Intracellular uptake of locally injected NDs by glial cells in the brain

**Fig. 1:**
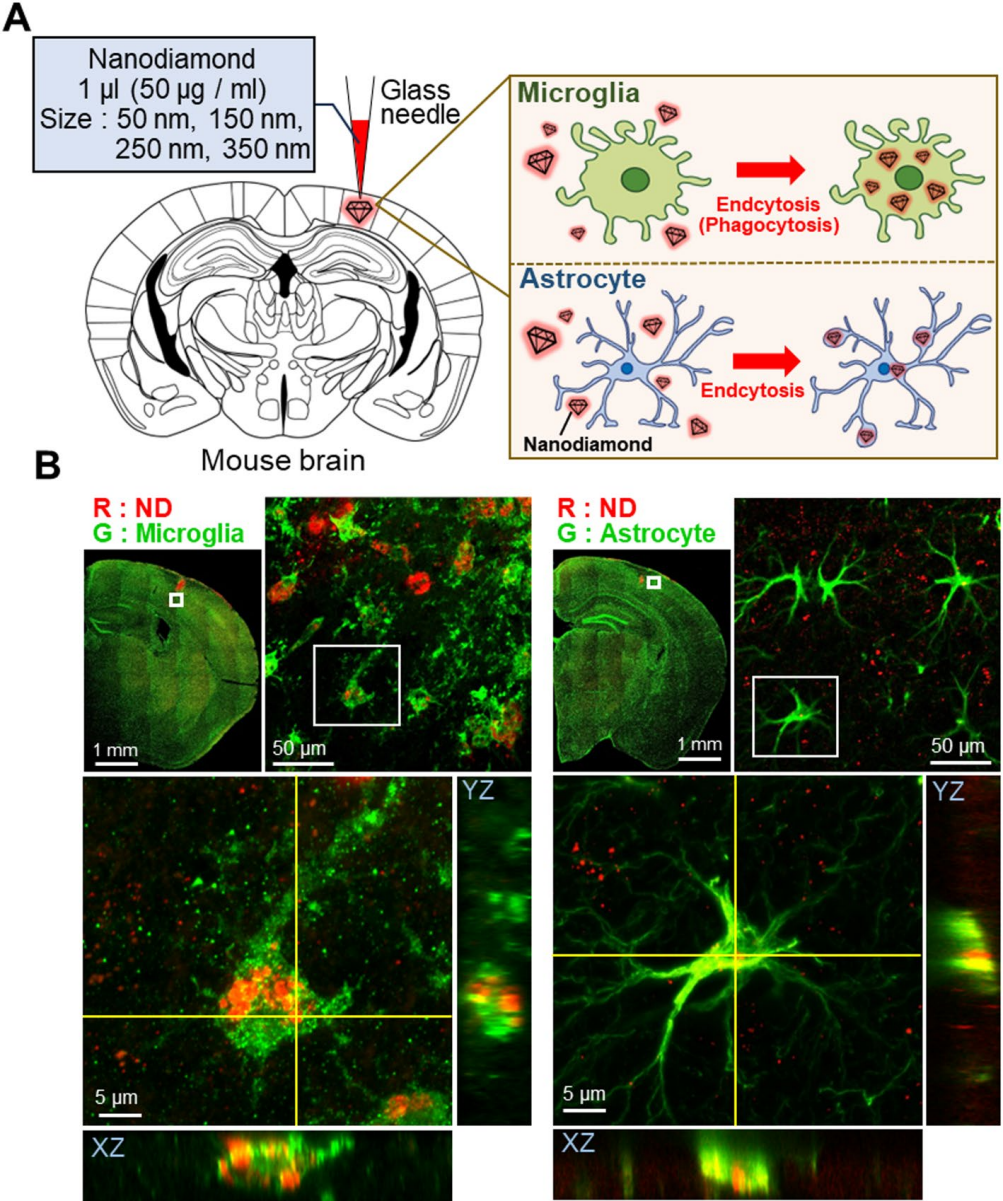
Immunofluorescence labeling of microglia and astrocytes. **A** Nanodiamonds (NDs) were locally injected into the brain parenchyma using a glass needle, and their phagocytosis by glial cells was examined. **B** Three-dimensional images of microglia and astrocytes that phagocytosed NDs demonstrate the uptake of each cell type. NDs are shown in red, while glial cells are represented in green

Four different ND sizes were prepared: 50 nm, 150 nm, 250 nm, and 350 nm, with each size surface-modified with COOH, HPG or HPG-COOH, resulting in a total of twelve types of NDs. These NDs were locally injected into the brain parenchyma using a fine glass micropipette, followed by immunostaining of glial cells after brain extraction (Fig. 1A). The fluorescence intensity of the NDs was carefully separated from brain tissue autofluorescence through spectral analysis. Figure 1B presents confocal microscopy images showing microglia and astrocytes along with intracellularly embedded NDs. Red-colored NDs were clearly observed within the cell bodies of microglia and astrocytes near the injection site. Figure 1B also contains projections along the xz and yz planes of a glial cell stack image, confirming the intracellular uptake of NDs.

### Intracellular uptake of NDs by microglia

**Fig. 2:**
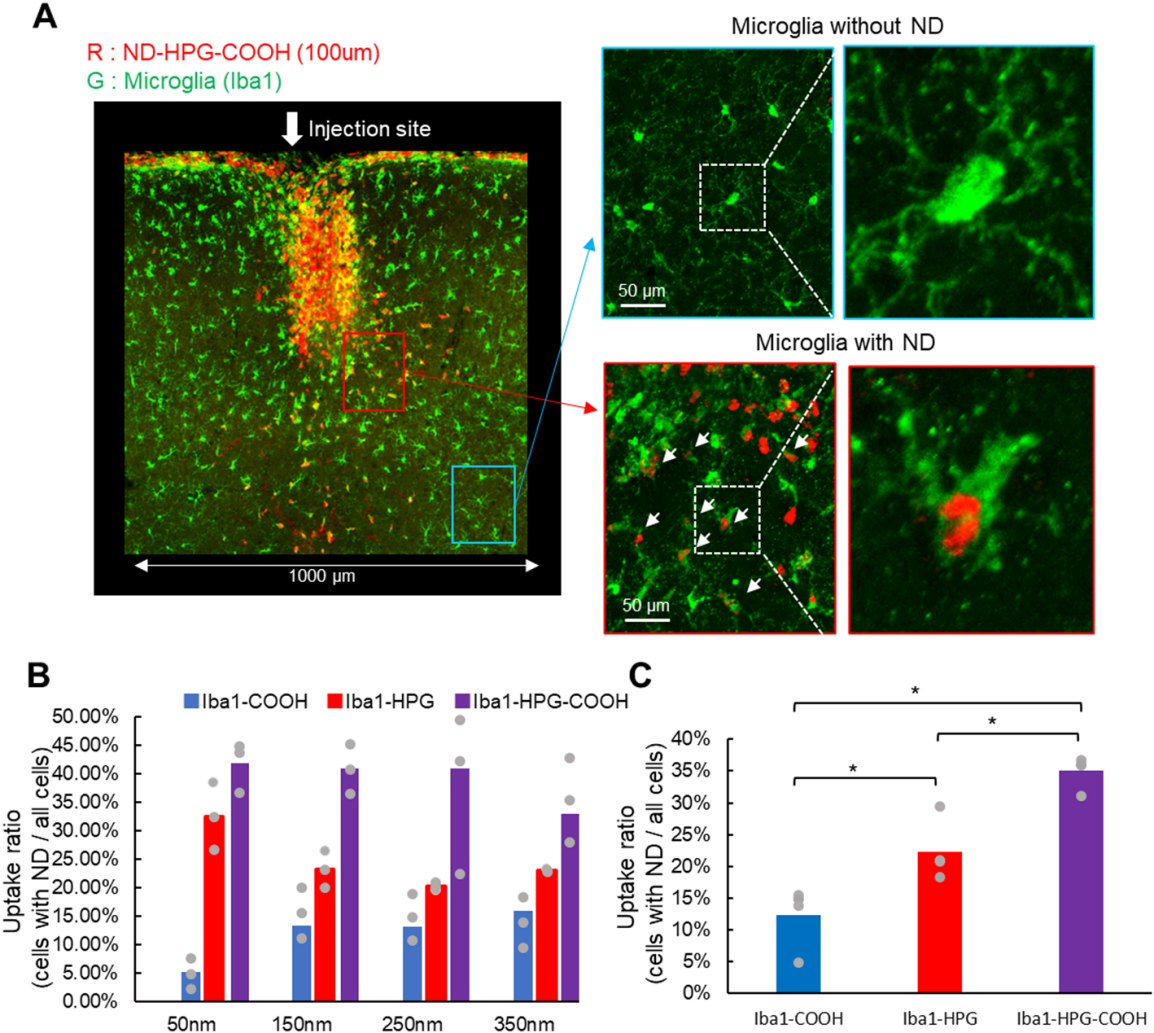
Quantification of ND uptake by microglia. **A** Microglia that phagocytosed NDs were counted within a 1 mm^2^ region surrounding the injection site. **B** The uptake rate of NDs by the microglia was compared for each combination of the different surface modifications (COOH, HPG, HPG-COOH) and sizes (50 nm, 150 nm, 250 nm, 350 nm). **C** Statistical analysis found that the NDs with the HPG-COOH surface modification exhibited the highest intracellular uptake rate regardless of size (*p < 0.001)

All microglia within a 1 mm^2^ area surrounding the injection site, including those that had phagocytosed NDs, were analysed (Fig. 2A and Table 1). As there were no strong size-dependent differences in intracellular uptake rates (Fig. 2B), the data were combined to analyse the effect of the three surface modifications (Fig. 2C). ANOVA showed that there was a significant difference between the results for the three surface modifications (*p* = 5.63 × 10^–11^, Bonferroni). Further analysis revealed that HPG-COOH modified NDs exhibited the highest cellular uptake rate, followed by COOH and HPG, with statistically significant differences between them (HPG-COOH vs. HPG, *p* = 2.45574 × 10^–5^; HPG vs. COOH, *p* = 4.34 × 10^–4^).

**Table 1:** Average number of microglia cells

COOH	50 nm	150 nm	250 nm	350 nm
Microglia with ND	20.5	54.5	70	40
Microglia without ND	383.5	307	389.5	268

HPG	50 nm	150 nm	250 nm	350 nm
Microglia with ND	155	76.5	69	71
Microglia without ND	323	254	323	271.5

HPG-COOH	50 nm	150 nm	250 nm	350 nm
Microglia with ND	92	148	88	78
Microglia without ND	233	261.5	275.5	194.5

**Fig. 3:**
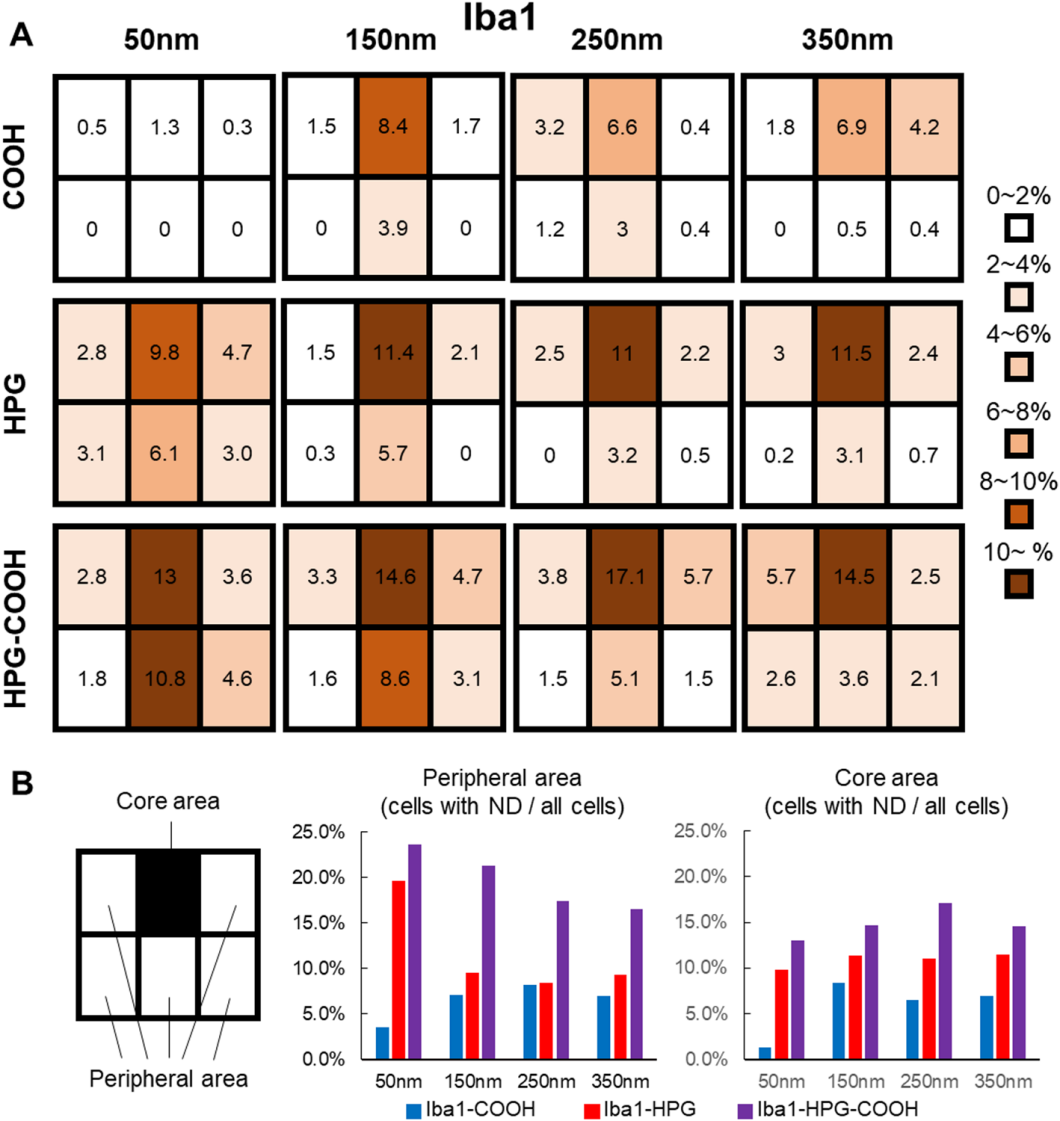
Cellular uptake rates of surface-modified NDs in the injection and surrounding areas. **A** A 1 mm^2^ region surrounding the injection site was divided into six quadrants, and the proportion of microglia that had taken up NDs was calculated for each region. The uptake rates were compared for NDs with different surface modifications (COOH, HPG, HPG-COOH) and sizes (50 nm, 150 nm, 250 nm, 350 nm) and visualized as a heat map. **B** The uptake rates of microglia were quantified for both the Core area (black rectangle) and the Peripheral area (white rectangles) for the three surface modifications and four different ND sizes

Next, the degree of nanodiamond dispersion from the injection site into the surrounding tissue and subsequent cellular uptake was evaluated by comparing the intracellular uptakes of microglia at the injection site and peripheral regions. Figure 3 illustrates the uptake rates of the NDs. HPG and HPG-COOH-modified NDs displayed high cellular uptake even in the peripheral region, suggesting greater dispersibility (Fig. 3A). In contrast, COOH-modified NDs exhibited a much lower uptake rate, and particularly so in the peripheral region.

### Intracellular uptake of NDs by astrocytes

**Fig. 4:**
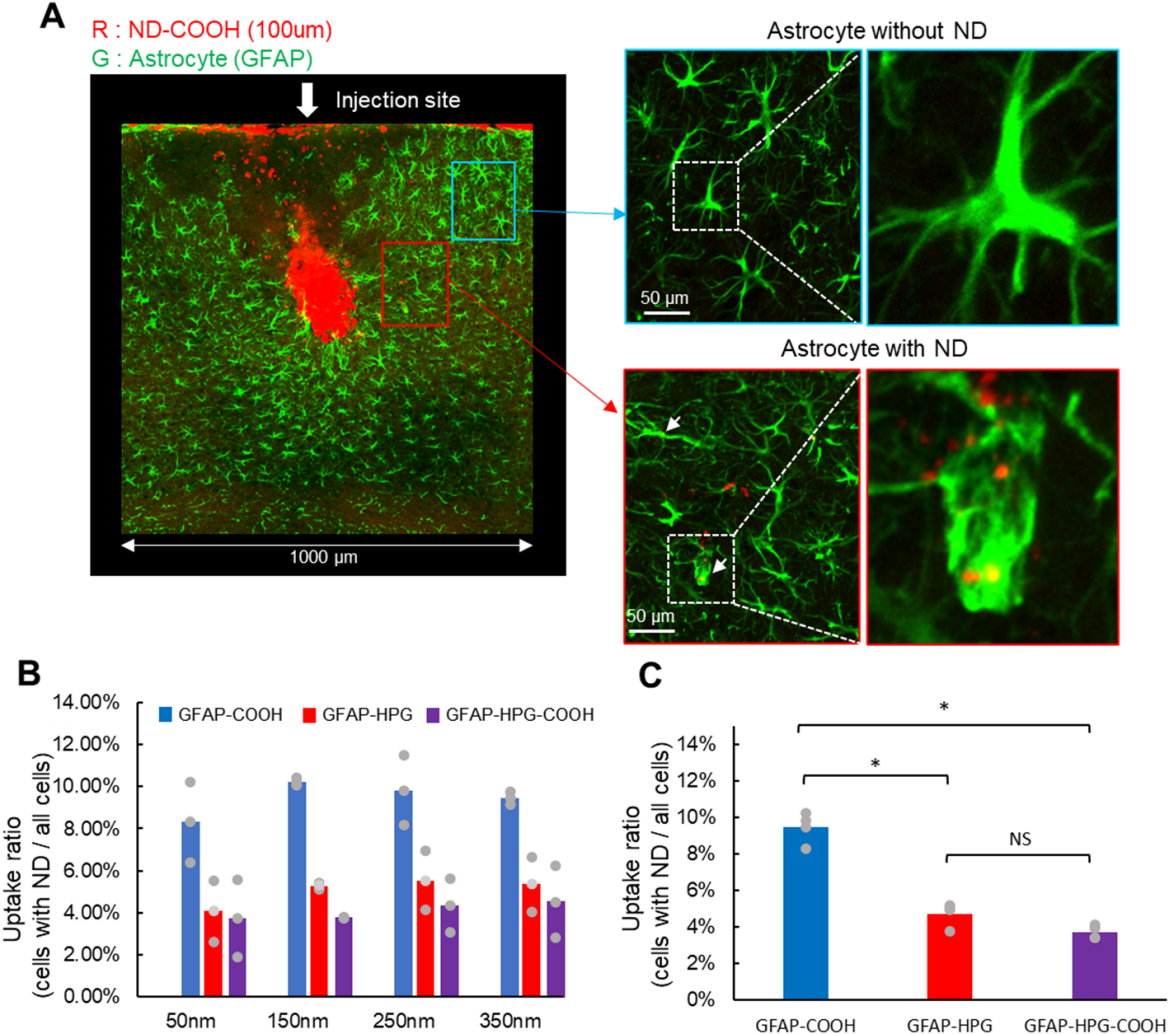
Quantification of ND uptake by astrocytes. **A** Astrocytes that phagocytosed NDs were counted within a 1 mm^2^ region surrounding the injection site. **B** The uptake rate of NDs by the astrocytes was compared for each combination of the surface modifications (HPG, COOH, HPG-COOH) and sizes (50 nm, 150 nm, 250 nm, 350 nm). **C** Statistical analysis found that the NDs with the COOH surface modification exhibited the highest intracellular uptake rate independent of size (*p < 0.001)

Astrocytes were fluorescently labeled with GFAP antibodies and imaged using confocal microscopy (Fig. 4A). Similar to microglia, astrocytes also showed no strong size-dependent differences in intracellular uptake rates (Fig. 4B and Table 2), so the data were combined to evaluate the effect of the three surface modifications (Fig. 4C). ANOVA showed that there was a significant difference between the results for the three surface modifications (*p* = 2.183 × 10^–13^, Bonferroni). Further analysis revealed that COOH-modified NDs exhibited the highest uptake rate (COOH vs. HPG, *p* = 8.37355 × 10^–9^; HPG vs. HPG-COOH, *p* = 0.11727).

**Table 2:** Average number of astrocyte cells

COOH	50 nm	150 nm	250 nm	350 nm
Astroglia with ND	25	35	27	29.33333
Astroglia without ND	284	308	259.5	283.6667

HPG	50 nm	150 nm	250 nm	350 nm
Astroglia with ND	13	17	17	21
Astroglia without ND	318	328	299	401

HPG-COOH	50 nm	150 nm	250 nm	350 nm
Astroglia with ND	9	11	10.5	10.5
Astroglia without ND	264.5	310.5	252.5	309.3333

**Fig. 5:**
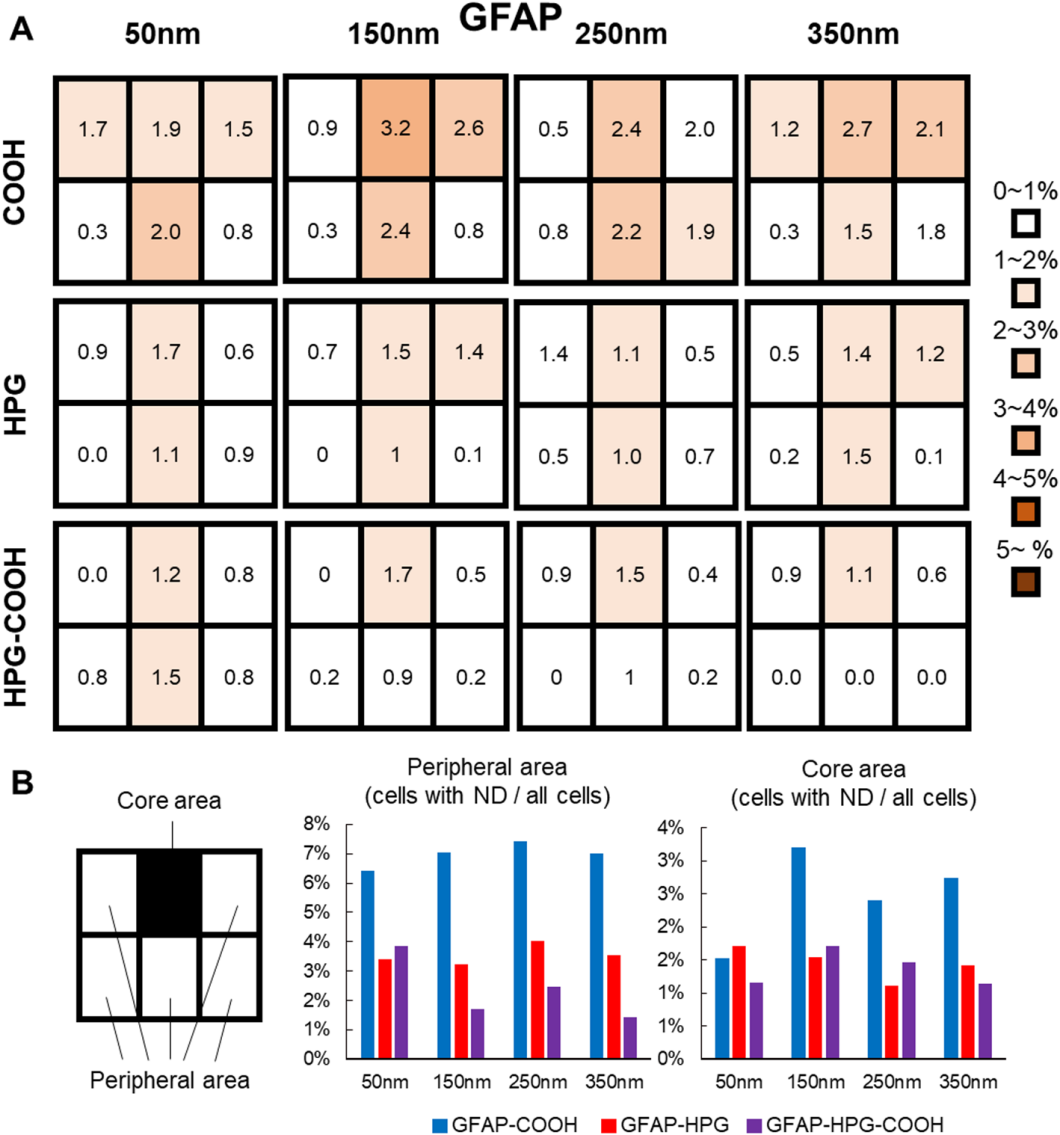
Cellular uptake rates of surface-modified NDs in the injection and surrounding areas. (**A**) 1mm^2^ region surrounding the injection site was divided into six quadrants, and the proportion of astrocytes that had taken up NDs was calculated for each region. The uptake rates were compared for NDs with different surface modifications (COOH, HPG, HPG-COOH) and sizes (50 nm, 150 nm, 250 nm, 350 nm) and visualized as a heat map. (**B**) The uptake rates of astrocytes were quantified in both the Core area (black rectangle) and the Peripheral area (white rectangles) for the three surface modifications and four different ND sizes

Figure 5 presents the uptake rate of NDs at the injection site and peripheral regions. No significant differences in uptake rate were observed across the different ND sizes or surface modifications. These findings suggest that astrocytes preferentially take up NDs that remain near the injection site.

## Discussion

NDs used as nano-quantum sensors are stable fluorescent materials with low biotoxicity, making them highly useful as tracers for life science research [1–3, 7]. Initially, the application of nano-quantum sensors in the life sciences focused on in vitro experimental systems, such as cultured cells. In recent years, there have also been some applications to in vivo biological tissues [8, 9]. However, to achieve the next step of in vivo intracellular applications, the development of techniques to reliably deliver NDs into cells within biological tissue is a crucial and urgent technical challenge. In this study, NDs with three types of surface modifications and four different sizes were locally injected into mouse brain parenchyma, and the intracellular uptake by microglia and astrocytes was quantitatively evaluated.

This study was designed with future in vivo applications of NDs in mind. The cerebral cortex was chosen as the target region because it offers excellent optical accessibility through a cranial window, allowing for imaging using wide-field one-photon or two-photon fluorescence microscopy. The cerebral cortex also has a functional relevance because it plays a critical role in sensory processing, neural signal transmission, and inflammatory responses [23, 24]. These characteristics make the cortex an ideal region for evaluating the distribution of NDs, and for potential future in vivo ND applications to study brain function and pathology.

### Microglial uptake of NDs

Microglia preferentially internalized HPG-modified NDs (HPG and HPG-COOH), (Fig. 2C). In comparison to the COOH-modified NDs, substantially more HPG-modified NDs were present in the peripheral area (Fig. 3). This suggests that NDs with HPG modification exhibited greater penetration into the brain tissue, leading to increased interactions with microglia in the peripheral region and, ultimately enhancing intracellular uptake. Previous in vitro studies of nanoparticle uptake by microglia have shown that the intracellular uptake rate depends on nanoparticle concentration and contact duration [15]. Our findings are consistent with those prior studies. It appears that NDs that disperse widely within the brain parenchyma have enhanced intracellular delivery to highly phagocytic microglia. Furthermore, research on macrophage cultures has shown that the shape of NDs influences uptake, with rounded NDs being internalized more efficiently than those with spiky structures [25]. If the microglial mechanism for intracellular uptake is similar to that of macrophages, it is possible that the improved uptake of NDs observed in this study was the result of increased roundness due to the surface modification.

In addition to the physicochemical properties of NDs, it is also important to consider the role of the in vivo environment. The injection of NDs using a fine glass needle may have induced mild local inflammation and activated microglia near the injection site. It is well known that activated microglia exhibit markedly increased phagocytic activity [26]. Therefore, the observed uptake of NDs may also reflect an enhanced phagocytic state of microglia in response to local inflammatory stimuli. To clarify these possibilities, future studies should incorporate immunohistochemical markers such as CD68 and IL-1β to evaluate the local activation state of microglia and assess its impact on ND uptake.

### Astrocytic uptake of NDs

In this study, we investigated whether astrocytic intracellular uptake of NDs differs depending on their size. Previous studies have reported that microglia and astrocytes target different structures for phagocytosis. Microglia primarily engulf neuronal cell bodies, whereas astrocytes preferentially phagocytose axons [12]. Given that axons are smaller than neuronal cell bodies, it might be expected that astrocytes would favor smaller NDs for uptake. However, in the present study, we did not observe a size-dependent difference in the uptake of NDs by astrocytes. A possible explanation for this, is that the NDs partially aggregated after in vivo administration (re Figs. 1B, 2A and 4A), and this aggregation may have diminished the effect of the original particle size within the brain. To clarify this issue, future studies could investigate the size distribution of ND aggregates formed in vivo.

A comparison of intracellular uptake for the three types of surface-modified NDs revealed that COOH-modified NDs were more effectively internalized by astrocytes than HPG-modified NDs. Astrocytes also exhibited a much lower uptake rate of HPG-modified NDs compared to microglia. However, the mechanism underlying this decreased uptake remains unclear. Based on the experimental data, it can be inferred that COOH-modified NDs tend to aggregate and remain near to the local injection site. It is also known that the phagocytic activity of astrocytes increases under inflammatory conditions [16]. In that case, astrocytes near the injection site may have been activated due to inflammation caused by tissue injury, leading to better uptake of COOH-modified NDs present in the vicinity. Conversely, HPG-modified NDs that dispersed away from the local injection site likely had a higher probability of encountering non-activated (low-phagocytic) astrocytes, and so there was a lower uptake of those NDs. That is, the inflammatory environment may be a significant modulator of ND internalization by astrocytes. In future studies, analysing the overlap between astrocyte activation markers and ND localization will be crucial to dissect the relative contributions of inflammation and ND surface properties. In summary, COOH-modified NDs appear to be the most suitable for intracellular delivery to astrocytes.

### Further remarks

The surface charge and hydrophilicity of NDs play a critical role in their interactions with the biological environment. Previous studies have shown that COOH modified ND typically exhibits a strong negative charge under physiological conditions [27–29]. Surface modification with HPG is expected to partially shield this charge and reduce nonspecific adsorption and aggregation [27]. Additionally, HPG–COOH, which introduces carboxyl groups at the termini of HPG, is presumed to carry a slight negative charge while retaining similar anti-adsorptive and anti-aggregative properties to unmodified HPG [27]. These physicochemical characteristics directly influence cellular uptake behaviors. HPG-modified NDs are known to resist protein corona formation due to their high hydrophilicity and electrical neutrality, thereby effectively evading phagocytosis [30]. In contrast, HPG–COOH-modified NDs can selectively bind to positively charged proteins through their negatively charged carboxyl groups, even in the presence of a protein-repellent HPG layer. This selective interaction may trigger recognition by cells, facilitating uptake via phagocytosis [31]. Furthermore, COOH-modified NDs without HPG shielding may interact with cell membranes not only via protein corona-mediated mechanisms, but also through direct adsorption due to exposed hydrophobic domains. Such interactions can promote cellular attachment and internalization, particularly in cell types that primarily rely on endocytic pathways [30]. Future work will involve physicochemical characterizations, including zeta potential measurements, to further elucidate how these surface modifications affect the in vivo behavior and cellular uptake of NDs.

The NDs utilized in this study, particularly the HPG- and HPG–COOH-modified NDs, are considered to be internalized primarily by glial cells via phagocytosis due to reduced membrane attachment. Additionally, since the NDs lack cell-specific targeting ligands, the uptake is presumed to occur under non-specific conditions. In the future, it may be possible to achieve more targeted intracellular uptake by modifying the ND surfaces with antibodies. Additionally, the method of injecting NDs into the brain parenchyma using a fine glass needle, as employed in this study, inevitably causes minor tissue damage and may induce inflammation. Therefore, developing techniques for delivering NDs to the brain via intravenous injection will also be an important future challenge.

This study elucidates the favorable cellular uptake characteristics of surface-modified NDs, but some long-term safety concerns regarding their metabolism and excretion remain critical. In this study, approximately 15 μg of NDs were administered into the mouse brain per animal, which is an extremely small amount compared to the systemic doses used in previous toxicological studies. For example, intravenous administration of detonation NDs at doses up to 250 mg/kg in mice did not result in observable toxicity over a 14-day period [32]. Similarly, other studies have demonstrated that NDs transiently accumulate in organs such as the liver and spleen, but are gradually cleared and generally well tolerated [28]. Given the low dose and localized administration in our study, the systemic impact of the NDs is thought to be negligible. Nevertheless, the long-term fate of NDs in brain tissue—including their metabolism, clearance, and aggregation—remains an important consideration. To clearly elucidate these aspects, future studies should include comprehensive evaluations of biodistribution, blood clearance, and excretion pathways. Furthermore, chronic exposure models and inflammation marker assays will be necessary to assess potential long-term toxicity and biocompatibility.

## Conclusion and future perspectives

In this study, we conducted experiments using NDs with various sizes and surface modifications to investigate the intracellular uptake by microglia and astrocytes in the brain. The experimental results indicate that HPG-modified NDs are optimal for cellular uptake by microglia, whereas COOH-modified NDs show the highest uptake efficiency in astrocytes. There was no clear dependence on size. Our findings show how efficient cellular introduction of NDs into microglia and astrocytes within the brain parenchyma can be performed. It is anticipated that the results will aid the measurement of ODMR signals from glial cells in the living brain.

## Supplementary Information

Below is the link to the electronic supplementary material.

Supplementary Material 1

## Data Availability

Data is provided within the manuscript or supplementary information files.
