# Supplementary material for: Investigating size and surface modification to optimise the delivery of nanodiamonds to brain glial cells
Source: Discov Nano. 2025 Aug 20;20(1):143. doi: 10.1186/s11671-025-04335-2 (PMC12367616; doi:10.1186/s11671-025-04335-2)
Supplement: Supplementary file 1 — Supplementary Material 1 [file 11671_2025_4335_MOESM1_ESM.docx]

Supporting Information

Investigating Size and Surface Modification to Optimise the Delivery of Nanodiamonds to Brain Glial Cells

Manami Takahashi^1 ‡^, Ayaka Takada ^1,2‡^, Chihiro Suzuki^2,3^, Kiichi Kaminaga^3^, Masaki Yoshioka1^4^, Mariko Handa^1,2^, Jeff Kershaw^5^, Hiroshi Abe^6^, Takeshi Ohshima^6^, Ryuji Igarashi^3^*, Hiroyuki Takuwa^1,2^*

^1^ Quantum Neuromapping and Neuromodulation Team, Institute for Quantum Life Science, National Institutes for Quantum Science and Technology, Chiba, JP 263-8555

^2^ Department of Quantum Life Science, Graduate School of Science and Engineering, Chiba University, Chiba, JP 263-0022

^3^ Future Quantum Sensors Team, Institute for Quantum Life Science, National Institutes for Quantum Science and Technology, Chiba, JP 263-8555

^4^ Department of Neurological Surgery, Graduate School of Medicine, Chiba University, Chiba, JP 260-8677

^5^ Department of Molecular Imaging and Theranostics, Quantum Life and Medical Science Directorate, National Institutes for Quantum Science and Technology, Chiba, JP 263-8555

^6^ Quantum Materials and Applications Research Center, Takasaki Institute for Advanced Quantum Science, National Institutes for Quantum Science and Technology, Takasaki, JP 370-1292

^‡^These authors contributed equally

Corresponding Author

*Hiroyuki Takuwa

Quantum Neuromapping and Neuromodulation Team, Institute for Quantum Life Science, Chiba, National Institutes for Quantum Science and Technology, JP 263-8555

Phone: +81-43-206-3425

E-mail: [takuwa.hiroyuki@qst.go.jp](mailto:takuwa.hiroyuki@qst.go.jp)

* Ryuji Igarashi

Future Quantum Sensors Team, Institute for Quantum Life Science, National Institutes for Quantum Science and Technology, Chiba, JP 263-8555

Phone: +81-043-206-4134

E-mail: [igarashi.ryuji@qst.go.jp](mailto:igarashi.ryuji@qst.go.jp)


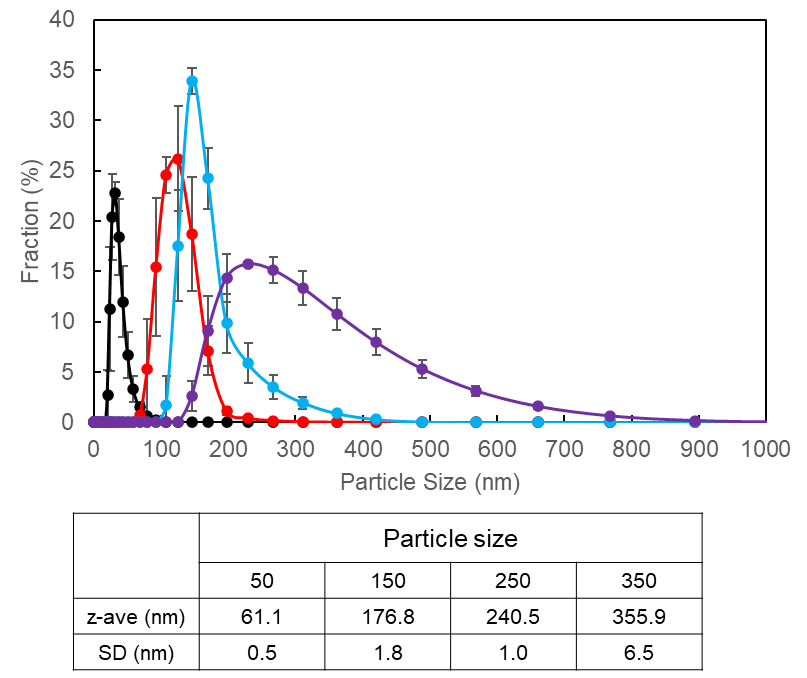


**Figure S1.** The Dynamic Light Scattering (DLS) results for nanodiamonds with various surface chemical modifications at 50, 150, 250, and 350 nm are presented. 50nm is represented by a black line, 150 nm by a red line, 250 nm by a blue line, and 350nm by a blue line.


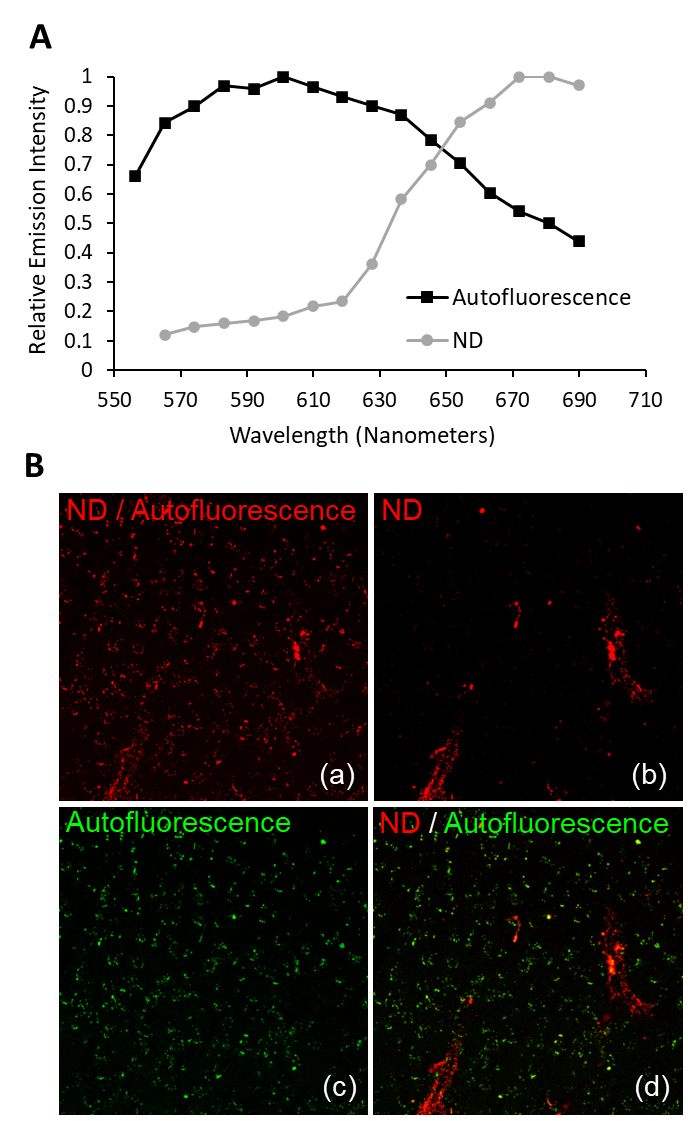


**Figure S2.** The removal of autofluorescence in a mouse brain that was injected with nanodiamonds and fixed. Spectral data for autofluorescence and nanodiamond of the as obtained with the LSM980 (Carl Zeiss). (B, a) In images using conventional main beamsplitters in a traditional confocal microscope configuration. The NDs and autofluorescence are both shown in red. (B, b and c) The successful separation of nanodiamonds and autofluorescence using spectral imaging and linear unmixing coupled with main beamsplitter enhancement is illustrated. NDs and autofluorescence are shown as red and green respectively. (B, d) Combined image of (B, b) and (B, c).
